# Prevalence of virulence factors in *Staphylococcus intermedius *isolates from dogs and pigeons

**DOI:** 10.1186/1746-6148-2-4

**Published:** 2006-01-26

**Authors:** Keiko Futagawa-Saito, William Ba-Thein, Naomi Sakurai, Tsuguaki Fukuyasu

**Affiliations:** 1 Department of Animal Health 2, School of Veterinary Medicine, Azabu University, 1-17-71 Fuchinobe, Sagamihara, Kanagawa, 229-8501, Japan; 2 Department of Molecular Microbiology/Immunology, Faculty of Allied Health Sciences, Thammasat University, Rangsit Campus, Pathum-thani, 12121, Thailand; 3 Center for Medical Sciences, School of Health Sciences, Ibaraki Prefectural University of Health Sciences, 4669-2 Ami, Inashiki, Ibaraki, 300-0394, Japan

## Abstract

**Background:**

*Staphylococcus intermedius *has been isolated from healthy dogs and pigeons as well as diseased dogs. Similar to *Staphylococcus aureus, S. intermedius *is known to carry many virulence factors but most of these factors remain to be studied. In this study, we examined 106 *S. intermedius *isolates (44 dog isolates and 62 pigeon isolates) for their hemolytic activity, biofilm formation, protease activity, and clumping factor and protein A production.

**Results:**

Forty-three dog isolates (97.7%) and all pigeon isolates were hemolytic on sheep RBCs with a mean hemolytic titer of 336.7 and 47.32, respectively, whereas 43 dog isolates (97.7%) and 11 pigeon isolates (17.7%) exhibited a significant difference in their hemolytic activity on rabbit RBCs with a mean hemolytic titer of 11.04 and 3.76, respectively (p < 0.0005). The mean biofilm formation activity for dog isolates was 0.49, which was significantly higher than that (0.33) for pigeon isolates (p < 0.0005). Twenty-four dog isolates (54.5%) and 11 pigeon isolates (17.7%) were protease positive. Twenty-four dog isolates (54.5%) were clumping factor- and protein A- positive.

**Conclusion:**

*S. intermedius *strains carrying the virulence factors examined in this study were more prevalent in dogs than pigeons.

## Background

*Staphylococcus intermedius *is found in a wide range of animal species including pigeons and dogs. It is recognized as normal flora of dogs and pigeons [1] and a principal causative agent of skin infections, mainly pyoderma, in dogs. It has been reported that the isolation frequency of *S. intermedius *from pigeons was two times higher than that from dogs [1] and that there is a genotypic diversity between *S. intermedius *isolates from dogs and pigeons [2-4].

Similar to *Staphylococcus aureus*, *S. intermedius *produces many virulence factors such as protease, coagulase, clumping factor, enterotoxins, exfoliative toxin, leukotoxin, and alpha and beta hemolysins [1,2,5-8]. It has been reported that the enterotoxin- and leukotoxin-producing *S. intermedius *strains are more prevalent in dogs than pigeons [1,2].

Biofilm formation by *S. aureus *strains isolated from bovine mastitis has been reported [9]. Biofilm formation is considered to be one of the virulence factors in Staphylococci, which helps Staphylococci adhere to its target tissues, mainly implants and other foreign body materials, through adhesive mechanisms [10,11]. Microcolonies encased in extracellular polysaccharide of biofilm are protected from antimicrobial agents [12]. The biofilm formation in *S. intermedius *has not yet been investigated.

In this study, we have examined *S. intermedius *isolates from dogs and pigeons with regards to their hemolytic activity, biofilm formation, protease activity, and clumping factor and protein A production.

## Results

### Clumping factor and protein A production

Twenty-four dog isolates (24/44, 54.5%) and none of the pigeon isolates were positive for clumping factor and protein A.

### Protease production

Protease production was significantly higher in dog isolates (24/44, 54.5%) than pigeon isolates (11/62, 17.7%) (p < 0.0005, Fisher's exact test).

### Hemolytic activity

With the exception of one dog isolate whose hemolytic titer was <2, all *S. intermedius *isolates (105/106, 99.1%), including 43 dog isolates (43/44, 97.7%) and all pigeon isolates, showed hemolytic activity on sheep RBC, and 54 *S. intermedius *isolates (54/106, 50.9%), including 43 dog isolates (43/44, 97.7%) and 11 pigeon isolates (11/62, 17.7%), showed hemolytic activity on rabbit RBC. Using sheep RBC, the mean hemolytic titer for dog isolates was 336.7 and that for pigeon isolates was 47.32 (p < 0.0005, t test). On rabbit RBC, the mean hemolytic titer for dog isolates was 11.04 and that for pigeon isolates was 3.76. There was a significant difference between the means of hemolytic titer on rabbit RBC for dog and pigeon isolates (Table 1, p < 0.0005, t test).

### Biofilm formation

The range of biofilm formation activity for all *S. intermedius *isolates was 0.02 to 1.00. The mean of biofilm formation activity for dog isolates was 0.49, which was significantly higher than that (0.33) for pigeon isolates (Table 1, p < 0.0005, t test).

### Association between hemolytic activity and biofilm formation activity

The association between hemolytic activity on rabbit RBC and biofilm formation activity is shown in Table 2. Regardless of the origin, the isolates with positive hemolytic activity had the mean biofilm formation activity of 0.49, whereas the isolates with negative hemolytic activity had significantly less biofilm formation activity with the mean activity of 0.29 (p < 0.0005). In addition, there was a significant difference in biofilm formation activity between hemolysis-positive and -negative pigeon isolates (0.52 vs. 0.28, p < 0.0005). However, there was no significant difference in biofilm formation activity between dog and pigeon isolates that had positive hemolytic activity on rabbit RBC (p = 0.49).

## Discussion

*S. intermedius *isolates from dogs and pigeons have been reported to be genotypically distinguishable [2-4]. Here, we observed a difference in their virulence traits such as hemolytic activity, biofilm formation, protease activity, and clumping factor and protein A production.

Hemolytic activity of *S. intermedius *isolates from healthy and infected dogs and pigeons has been described previously [5,7,13]. The number of hemolysis-positive isolates in this study (99.1% on sheep RBC and 50.9% on rabbit RBC) is higher than that (88.2% on sheep RBC and 1.5% on rabbit RBC) in a previous study in which blood agar plate was used for assay [7]. This observed difference could be due to the fact that a sensitive microplate technique was used in our study to determine the hemolytic activity. On the other hand, the mean hemolytic titers on rabbit and sheep RBC for dog isolates were significantly higher than that for pigeon isolates. Since dog isolates also exhibited a very high leukotoxic activity in a previous study [2], cytotoxin-producing *S. intermedius *strains seem to be prevalent among dogs.

Biofilm-forming *S. epidermidis *and *S. aureus *isolates have been recovered from hospitalized patients and non-hospitalized people [14], and instruments of dialysis [15], and bovine mastitis [9], and food and food processing environments [16], respectively. We tested a large number of *S. intermedius *isolates for their biofilm formability. Biofilm formation was significantly higher in the isolates from dogs than pigeons. Bacteria in biofilms are generally resistant to environmental stress [17], antibiotics [12], and phagocytosis by macrophage [18]. Therefore biofilm-forming *S. intermedius *isolates from dogs may have the potential to cause opportunistic and biomaterial-related infections.

Alpha-hemolysin, which is hemolytic on rabbit RBC [19], has been shown to be required for cell-to-cell interactions during biofilm formation in *S. aureus *[20]. Likewise, the association between biofilm formation and alpha-hemolysin production in *S. intermedius *was also observed in this study as the number of hemolysis-positive isolates was significantly higher than that of the hemolysis-negative isolates among the biofilm-forming *S. intermedius *isolates.

The accessory gene regulator (*agr*) of a two-component regulatory system in *S. aureus *is implicated in biofilm formation and alpha-hemolysin production [21]. In *S. intermedius*, an *agr*-like locus has also been identified by PCR [22], but the alpha hemolysin (*hla*) gene has not been reported. Therefore, it is of interest to further examine the production and regulation of virulence factors in *S. intermedius *strains.

*S. aureus *clinical isolates produce a variety of extracellular proteases [23]. Several in-vitro studies have suggested that extracellular protease is an important virulence factor in *S. aureus *[24,25]. Clumping factor promotes binding of fibrinogen and fibrin to the bacterial cell surface [26], and is shown to act as a virulence factor in experimental septic arthritis in *S. aureus *[27]. *S. aureus *isolates from patients with Kawasaki disease produce high levels of protein A [28], which is reportedly associated with inflammation of lungs [29]. More than half of *S. intermedius *isolates from dogs in this study produced protease and they were clumping factor and protein A positive. It is interesting to note that the clumping factor- and protein A- positive *S. intermedius *were isolated only from dogs. It is not known if protease, clumping factor, and protein A are associated with pathogenesis of *S. intermedius *infections in animals, but the carriage of these virulence factors indicate the pathogenic potential of the isolates. Besides, the production of many virulence traits tested in this study are susceptible or dependent on in-vitro conditions and it should cautious in interpretation of the virulence properties of *S. intermedius *isolates.

## Conclusion

This study demonstrated that *S. intermedius *strains carrying tested virulence factors are more prevalent in dogs than pigeons.

## Methods

### Bacterial strains

*S. intermedius *isolates (n = 106), including 44 isolates from dogs and 62 isolates from pigeons, were used in this study. Isolation and identification of *S. intermedius *isolates were done as described previously [1]. *S. aureus *RN4220 [30] and *S. hyicus *JCM2423^T ^[5] were used respectively as a positive control and a negative control in the hemolytic activity assay. *S. epidermidis *ATCC35984 was used as a positive control in the quantitative assay of biofilm formation.

### Clumping factor and protein A assay

Simultaneous detection of clumping factor and protein A was performed as described previously by Essers *et al. *[31]. *S. intermedius *isolates were cultured on brain heart infusion agar plate (Becton, Dickinson and Company, MD, USA) for 18 h at 37°C. A mixture of one drop each of culture (approximately 10^8 ^cfu) and saline was mixed with PS latex (Eiken, Tokyo Japan). Agglutination that occurred within one minute while stirring was considered a positive reaction.

### Protease activity

Protease activity was determined on casein agar plates following the procedure described by Bjorklind *et al*. [32]. The production of protease was recognized as a clear zone or a broad zone of precipitation around the bacterial streak [32,33].

### Assay for hemolytic activity

Hemolytic assay was performed by the microplate method [19] using sheep and rabbit erythrocytes (RBCs). A culture supernatant of overnight-grown bacteria at 37°C in brain heart infusion broth (Becton, Dickinson and Company, MD, USA) was used. Two-fold dilutions of the culture supernatant in PBS (pH 7.0) containing 0.1% bovine serum albumin (BSA) (50 μl each) were mixed with 50 μl of 1% RBC in PBS in a 96-well microtiter plate. The microtiter plate was incubated at 37°C for 1 h with gentle shaking and, for sheep RBC, further incubated at 4°C for 1 h without shaking. The microtiter plate was centrifuged at 600 × *g *for 5 min. The hemolytic activity titer was defined as the inverse of the last dilution that caused complete hemolysis. The isolates with hemolytic titer ≥2 were considered positive for hemolytic activity.

### Quantitative assay for biofilm formation

The assay was performed as previously described [9,34] with some modifications. Bacteria were cultivated overnight in trypticase soy broth, TSB, (Becton, Dickinson and Company, MD, USA) containing 0.25% glucose. Each culture was diluted 1:200 in the same broth. The cell suspension (200 μl) was inoculated into each well of sterile 96-well polystyrene tissue culture plates (Becton Dickinson Labware, NJ, USA) and incubated at 37°C for 16 h. The wells were washed twice with 200 μl of PBS (pH 7.4) and stained with 100 μl of 0.1% safranin-O solution per well for 30 s. After removal of the staining solution, the wells were washed once again with PBS. Then, 100 μl of a 97% ethanol-3% ether solution was added to each well and mixed. The absorbance of the adherent biofilm was measured at 490 nm in a microplate reader (Model 680, BioRad, CA, USA) and the absorbance value was expressed as the biofilm formation activity. The results were reported after subtracting the reading for a blank (TSB plus 0.25% glucose, without bacterial cells) from the experimental readings. Each assay was performed in triplicate.

## Authors' contributions

KFS conceived of the study, carried out all the experimental work and drafted the manuscript. WBT participated in analysis and interpretation of data, and wrote the final manuscript. NS participated in analysis and interpretation of data. TF participated in the study design and coordination. All authors read and approved the final manuscript.

## Figures and Tables

**Table 1:** Hemolytic activity and biofilm formation activity of *Staphylococcus intermedius *isolates from dogs and pigeons

Animal (no. of isolates)	Hemolytic titer on sheep RBC (1/dilution)		Hemolytic titer on rabbit RBC (1/dilution)		Biofilm formation activity (absorbance value at 490 nm)	
			
	Range	Mean	Range	Mean	Range	Mean
Dogs (44)	<2–2048	336.7^a^	<2–32	11.04^b^	0.02–1.00	0.49^c^
Pigeons (62)	8–128	47.32^a^	<2–16	3.76^b^	0.04–0.79	0.33^c^

a pair, b pair and c pair : p < 0.0005 (t test)

Note: Absorbance value at 490 nm is reported as biofilm formation activity

**Table 2:** Association between hemolytic activity on rabbit RBC and biofilm formation activity in *Staphylococcus intermedius *isolates from dogs and pigeons

Hemolytic activity on Rabbit RBC	Animal (no. of isolates)	Biofilm formation activity^c ^(absorbance value at 490 nm)	
		
		Range	Mean
Positive^a^	Dogs (43)	0.02–1.00	0.48^d^
	Pigeons (11)	0.22–0.71	0.52^d,e^

Total	(54)	0.02–1.00	0.49^f^

Negative^b^	Dogs (1)	0.71	0.71
	Pigeons (51)	0.04–0.79	0.28^e^

Total	(52)	0.04–0.79	0.29^f^

^a^Hemolytic activity ≥2; ^b^Hemolytic activity <2; ^c^after subtracting the absorbance value of blank (TSB with 0.25% glucose); d pair: p = 0.49; e pair and f pair: p < 0.0005
